# Premature primary tooth eruption in cognitive/motor-delayed ADNP-mutated children

**DOI:** 10.1038/tp.2017.27

**Published:** 2017-02-21

**Authors:** I Gozes, A Van Dijck, G Hacohen-Kleiman, I Grigg, G Karmon, E Giladi, M Eger, Y Gabet, M Pasmanik-Chor, E Cappuyns, O Elpeleg, R F Kooy, S Bedrosian-Sermone

**Affiliations:** 1 The Lily and Avraham Gildor Chair for the Investigation of Growth Factors, The Elton Laboratory for Neuroendocrinology, Department of Human Molecular Genetics and Biochemistry, Sackler Faculty of Medicine, Tel Aviv, Israel; 2 Sagol School of Neuroscience and Adams Super Center for Brain Studies, Tel Aviv University, Tel Aviv, Israel; 3 Department of Medical Genetics, University and University Hospital of Antwerp, Antwerp, Belgium; 4 Department of Anatomy and Anthropology, Sackler Faculty of Medicine, Sagol School of Neuroscience, Tel Aviv University, Tel Aviv, Israel; 5 The Bioinformatics Unit, George S. Wise Faculty of Life Sciences, Tel Aviv University, Tel Aviv, Israel; 6 Monique and Jacques Roboh Department of Genetic, Hadassah Hebrew University Medical Center, Jerusalem, Israel; 7 ADNP Kids Research Foundation, Brush Prairie, WA, USA

## Abstract

A major flaw in autism spectrum disorder (ASD) management is late diagnosis. Activity-dependent neuroprotective protein (*ADNP*) is a most frequent *de novo* mutated ASD-related gene. Functionally, ADNP protects nerve cells against electrical blockade. In mice, complete Adnp deficiency results in dysregulation of over 400 genes and failure to form a brain. Adnp haploinsufficiency results in cognitive and social deficiencies coupled to sex- and age-dependent deficits in the key microtubule and ion channel pathways. Here, collaborating with parents/caregivers globally, we discovered premature tooth eruption as a potential early diagnostic biomarker for ADNP mutation. The parents of 44/54 ADNP-mutated children reported an almost full erupted dentition by 1 year of age, including molars and only 10 of the children had teeth within the normal developmental time range. Looking at Adnp-deficient mice, by computed tomography, showed significantly smaller dental sacs and tooth buds at 5 days of age in the deficient mice compared to littermate controls. There was only trending at 2 days, implicating age-dependent dysregulation of teething in Adnp-deficient mice. Allen Atlas analysis showed *Adnp* expression in the jaw area. RNA sequencing (RNAseq) and gene array analysis of human ADNP-mutated lymphoblastoids, whole-mouse embryos and mouse brains identified dysregulation of bone/nervous system-controlling genes resulting from ADNP mutation/deficiency (for example, *BMP1* and *BMP4*). *AKAP6*, discovered here as a major gene regulated by ADNP, also links cognition and bone maintenance. To the best of our knowledge, this is the first time that early primary (deciduous) teething is related to the ADNP syndrome, providing for early/simple diagnosis and paving the path to early intervention/specialized treatment plan.

## Introduction

Autism spectrum disorders (ASDs) and related developmental conditions are typically diagnosed between 2 and 4 years of age;^1^ however, in many instances the cause remains elusive. Activity-dependent neuroprotective protein (ADNP) was discovered in the Gozes laboratory^2^ as a protein-protecting nerve cells against electrical blockade.^3^ Recent studies identified ADNP as a regulator of axonal transport,^4^ dendritic spine plasticity^5^ and autophagy.^6^ Complete Adnp deficiency in mice results in inability to form a brain.^7^ ADNP functions at the cytoplasm/synapse and the cell nucleus^8^ constituting a part of the essential chromatin-remodeling complex, SWItch/Sucrose Non-Fermentable (SWI/SNF).^9^ During embryonic development, Adnp regulates over 400 genes crucial for brain formation/organ development.^10^ Throughout postnatal brain maturation and adulthood, Adnp regulates multiple key genes associated with synaptic transmission including tubulin, ion channel and autophagy-controlling genes in an age/sex/genotype-dependent manner.^4^

Recent findings (Kooy's laboratory) place ADNP as one of the most frequent *de novo* mutated ASD genes, defined as the ADNP syndrome and also known as the Helsmoortel-Van der Aa syndrome.^11, 12, 13^ Notably, an original observation in the ADNP syndrome was facial dysmorphisms^11^ that may be associated with development of dentition and craniofacial skeleton.^14^

Many regulatory mechanisms that are involved in dentition are also active in other developmental processes. This explains certain syndromes resulting in failed or delayed dentition.^15^ The mechanisms of tooth eruption^16^ and orthodontic tooth movement^17^ involve different regulatory mechanisms on the occlusal and the apical sides of the erupting tooth. On the occlusal side, osteoclast differentiation is stimulated, leading to the development of an eruption canal, a process in which macrophages and matrix metalloproteases have an important role. On the apical side, runt-related transcription factor 2 (RUNX2) and the bone morphogenetic protein 2 (BMP2) are suggested to be critical for the deposition of trabecular bone.^18^ Cytokines and chemokine have an important role, for example, inactivation of interleukin 11 signaling causes premature closure of cranial sutures coupled to delayed tooth eruption.^19^

To the best of our knowledge and as obvious from the introduction above, no disease was previously associated with early deciduous dentition, except for natal teeth (present at birth), and 'neonatal teeth' that erupt within the first month of life^20^ and one limited case report.^21^

Here, observing 54 ADNP-mutated children worldwide, we have discovered premature teething as a potential early diagnostic biomarker for ADNP mutation related to ASD. Eighty-one percent of the screened ADNP-mutated children reported fully erupted dentition by 1 year of age, including molars, and only 10 of the children had teeth within the normal developmental time range. Computed tomography analysis in mice showed dysregulated tooth eruption in the Adnp-deficient mice compared to controls.

Deep insight into our mouse embryo gene expression data suggested an interaction of ADNP with bone/tooth development. Thus, in *Adnp* knockout embryos, Affymetrix (Santa Clara, CA, USA) complete gene array showed that *Bmp1* was downregulated compared to *Adnp*-intact embryos.^10^ Here, our RNA sequencing (RNAseq) results comparing three ADNP-mutated lymphoblastoid cell lines (LCLs, derived from ADNP-mutated children) with a non-mutated cell line identified multiple functional changes in the expression of bone morphogenesis genes.

Interestingly, the same developmental factors regulate nervous system development as tooth development, namely the BMPs.^22^ Given that complete *Adnp* deficiency results in neural tube closure defect, it is conceivable that tooth deregulation may be associated with Adnp deficiency, as observed here.

Together, important data presented here, for we believe the first time, showed that early simple diagnosis of ADNP-mutated children and related ASD cases might be feasible by careful monitoring of early deciduous teething.

## Materials and methods

### ADNP kids research foundation

To provide information/advocacy/emotional support to families worldwide (for example, Gozes *et al.*^12^), an ADNP Syndrome Parents Group was established on the social networking site Facebook (like other support groups, for versatile diseases;^23^ www.ADNPkids.com). The members are in close contact with researchers both collecting information about the mutated children to further the understanding of the syndrome and help the children and families. Currently, over 75 families are assembled, connected through the original set up of ADNP syndrome (http://www.omim.org/entry/611386). Here, we include results of a questionnaire about tooth development that has been posted on ADNP parent website. The sample size was determined by the number of parents willing to participate on the ADNP parent website. The informal questionnaire included questions regarding time of deciduous tooth eruption, tooth eruption state with reference to molars at 1 year of age, corroboration by tending dentists and photographs of children under informed consent to publish. Other questions addressed permanent tooth eruption time, again with reference to molars and further questions addressed autism diagnosis. Written informed consent for inclusion and consent for the publication of photographs in the study were obtained from all patients. Studies were approved by the Tel Aviv University Ethics Committee.

### Micro-computed tomography

New-born mice mandibles and children dry shed teeth were scanned using a μCT50 system (Scanco Medical, Brüttisellen, Switzerland) at 17.2 and 34.4 μm resolution, respectively, with 90 kV energy, 200 μA and 1000 projections of 1 s. Morphometric evaluation of the mineralized tissue was performed using Scanco UCT_EVALUATION v6.6. Remaining mouse tissue samples were subjected to genotype analysis.^7, 24^

### Tooth eruption in Adnp^+/−^ mice

All procedures involving animals were approved by the Animal Care and Use Committee of Tel Aviv University and the Israeli Ministry of Health. Adnp^+/−^ mice on a mixed C57BL and 129/SvJ background, a model for cognitive impairments,^7^ were housed in a 12 h light/12 h dark cycle facility, with free access to rodent chow and water.^7, 24^ To allow continuous breeding and excellent progeny, an imprinting control region outbred line was used.^25^ For precise evaluation of tooth eruption, 2- and 5-day-old mouse pups (males and females) were decapitated and their heads were fixed in 4% paraformaldehyde, transferred to phosphate-buffered saline after 48 h and subjected to μCT analysis (above). The dental sac was separated from the surrounding tissues by manual contouring (for the incisors, only in the part inside the alveolar bone) and the mineralized tissue by global thresholding. No randomization was applied. The investigators were not blinded to the genotype of the pups. Animal group size was chosen to allow statistical assessments.

### Epstein–Barr virus-transformed human LCLs

A representative LCL from healthy adult donors was obtained from the National Laboratory for the Genetics of Israeli Populations (NLGIP; http://nlgip.tau.ac.il), Tel Aviv University. Two ADNP-mutated LCLs were purchased from the Simon Simplex Collection, SSC04121=ADNP (protein) p.Lys408Valfs*31 and SSC08311= p.Tyr719* (ref.^11^) and one was generated from peripheral blood lymphocytes donated by consenting patient and guardians/physicians (OE). Additional validation LCLs were obtained from the Kooy's laboratory (p.Gln40* p.Ser404* p.Lys408Valfs*31; p.Leu831Ilefs*81; and p.Asn832Lysfs*80). All tissue-culture reagents were purchased from Biological Industries (Beit-Haemek, Israel). Cells were maintained in optimal growth conditions as described.^26^ In short, cells were kept at a temperature of 37 °C with 99% humidity and 8% CO_2_ and maintained in Roswell Park Memorial Institute (Cat: 01-100-1A) medium supplemented with 10% fetal bovine serum (Cat: 04-007-1A) and antibiotics (1% penicillin–streptomycin; 1% l-glutamine (Cat: 03-020-1B; 03-031-1B). Cells were tested negative for mycoplasma, using the EZ-PCR Mycoplasma Test Kit (Cat: 20-700-20). Studies were approved by the Tel Aviv University Ethics Committee.

### RNAseq and bioinformatics

RNAseq and bioinformatics were performed as previously described on the LCL lines.^4^ The data have been uploaded to GEO, GSE81268. Further details are in the Supplementary Information. Functional enrichment was performed using DAVID tool.^27^ Network analysis was performed using GeneMania tool^28^ and the STRING server.^29^

### RT-PCR verification

RNA was extracted using TRI reagent (T9242, Sigma-Aldrich, St. Louis, MO, USA). At the time of extraction, cell confluence was ~70–80%. Reverse transcription was performed on 500 ng RNA from each LCL. The reaction was carried out using the qScript cDNA Synthesis Kit (Cat: 25-047-100, Quanta Biosciences, Gaithersburg, MD, USA). Quantification of gene expression was performed on 1ng μl^−1^ cDNA per well, 200 nM of each primer with KAPA SYBR FAST qPCR Kit (Cat: KK4602, Kappa Technologies, Woburn, MA, USA) using QuantStudio 12 Flex System (Applied Biosystems, Foster City, CA, USA) for 40 cycles. Gene expression was normalized between different samples based on values of *TATA-box* expression. Results are presented as 2^−ΔCT^. All real-time polymerase chain reaction (PCR) reactions were performed in triplicates.

PCR primers:

BMP1: 5′- GCAGTCTACGAAGCCATCTG -3′

3′- ATGAGGGTGCTGCTCTCACT -5′

BMP4: 5′- GACTTCGAGGCGACACTTCT -3′

3′- TCCAGATGTTCTTCGTGGTG -5′

AKAP6: 5′- CTCACAAAGCAGGACTGAAGG -3′

3′- CCTTCCTCATCCTCCACAGA -5′

TATA-box: 5′- AGTTCTGGGATTGTACCGCA -3′

3′-TGTGCACACCATTTTCCCAG-5′.

### Statistical analysis

All results are shown as means±s.e.m. Statistical analyses was conducted using Student's *t*-test with Excel (Microsoft, Redmond, WA, USA). Outliers were excluded using the Graphpad outlier calculator (https://graphpad.com/quickcalcs/Grubbs1.cfm). Statistical significance for all tests was set to *P*-value⩽0.05. For RNAseq, statistical analysis was performed as previously described.^4^

## Results

### Early deciduous tooth eruption

A survey of 54 families with various ADNP mutations (Table 1) showed early deciduous teething among 81% of the children with an almost fully erupted dentition including molars by 1 year of age. Interestingly, in some cases, several teeth were reported to erupt at once in the children with ADNP mutations. In all, 10/54 surveyed children had teeth within the 'normal' range.^30, 31^ An additional survey of 18 healthy siblings did not identify any case of early deciduous tooth eruption, nor full dentition by 1 year of age. Between 95 and 100% of the surveyed ADNP syndrome children were motor/cognitively delayed, 85% were either diagnosed or had autistic traits and of the remaining children 2 were too young to diagnose.

**Figure 1:**
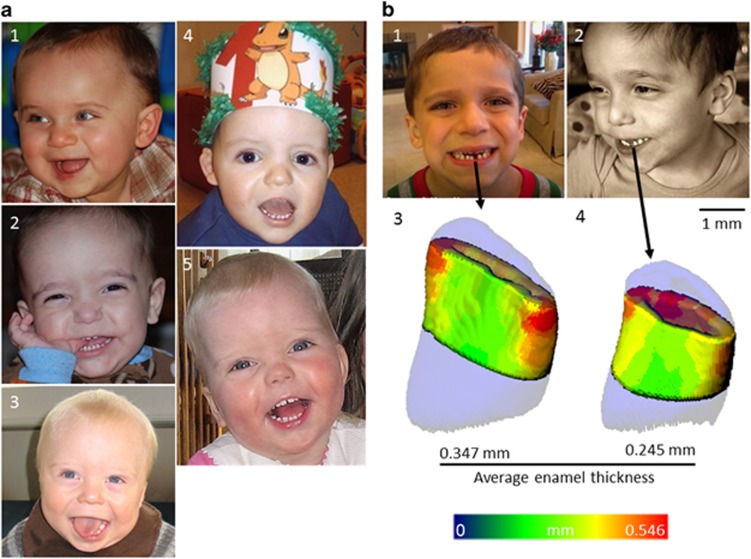
Pictures of children and teeth. (**a**) Facial pictures: (a_1_) Picture of a control ADNP-intact boy at 11 months. (a_2_) Picture of full erupted dentition at 11 months of age. Patient mutation p.Leu349Argfs*47 (Table 1 (ref. 12)) at 11 months of age (2 weeks before his first birthday) with erupted first molars. Teeth erupted around 9 months of age. C_3_ p.Lys831 llefs*81 (Denmark on Table 1), (a_4_), 1-year-old boy, mutation, p.Lys831 llefs*81, same mutation (Belgium on Table 1, also denoted patient 4 (ref. 11)), (5) 1-year-old girl, mutation, p.Asn832Lysfs*80 (second girl from the Netherlands). (**b**) Pictures and shed teeth after micro-computed tomography (μCT). (b_1_) (5-year-old) and (b_2_; 4-year-old) pictures of the twins a_1_ and a_2_, respectively, at the time of first tooth shed. (b_3_ and b_4_) μCT images of the shed tooth (left central lower incisor) in b_1_ and b_2_, respectively. Warm colors represent greater thickness of the enamel. ADNP, activity-dependent neuroprotective protein.

Example pictures of children at 1 year of age are shown (Figure 1a1–5). Figure 1a1 shows the ADNP-intact (11-month-old) twin brother of a boy with ADNP syndrome,^12^ shown in Figure 1a2, at the same age. The differences are striking, as also noted by the dentist, with the ADNP child presenting almost full erupted dentition at 1 year of age (Figure 1a2). The dentist record noted that the patient, presented on 19 March 2009 at 13 months of age, had deciduous incisors, canines and first molars, totaling 16 erupted teeth. This finding was a representative of the parent's (SBS) concern of premature tooth eruption. At the same age, the ADNP-intact twin brother had barely 8 erupted teeth. Figure 1a3–5 shows pictures of three additional ADNP-mutated children, at about 1 year of age, all exhibiting full erupted dentition, regardless of the specific ADNP mutation (that is, spanning the ADNP cDNA/protein, see also Table 1).

### Early deciduous tooth-shedding and altered permanent tooth eruption

Similar to the results described for early deciduous tooth eruption, first tooth-shedding occurred on 23 July 2013 in the ADNP-intact child depicted in Figure 1a_1_ as a baby and on Figure 1b_1_ at the time of tooth-shedding (~5 years of age). His twin brother (ADNP-mutated) showed first tooth-shedding on 15 June 2012, a year earlier, at 4 years of age (Figure 1b_2_). The shed central lower incisors were subjected to μCT, and the mutated child tooth was smaller and had a thinner enamel (0.245 versus 0.347 mm mean thickness, 40% difference, Figures 1b3–4).

Additional reports implicated early second permanent molar eruption in two girls, in whom expected eruption time was ~12 years (http://www.ada.org/~/media/ADA/Publications/Files/patient_58.ashx), one at 8.5 and one at 10 years of age (both share a mutation, p.Tyr719*). Another boy with p.Lys274Asnfs*31 mutation had two of his first permanent molars at ~4 years of age (expected eruption time, 6–7 years of age). In contrast, a young man with a p.Tyr719* has his third molars (wisdom tooth) erupting at 24 years of age, which is a little late compared to the published expected eruption between 17 and 21 years of age.

### Tooth eruption in Adnp^+/−^ mice

**Figure 2:**
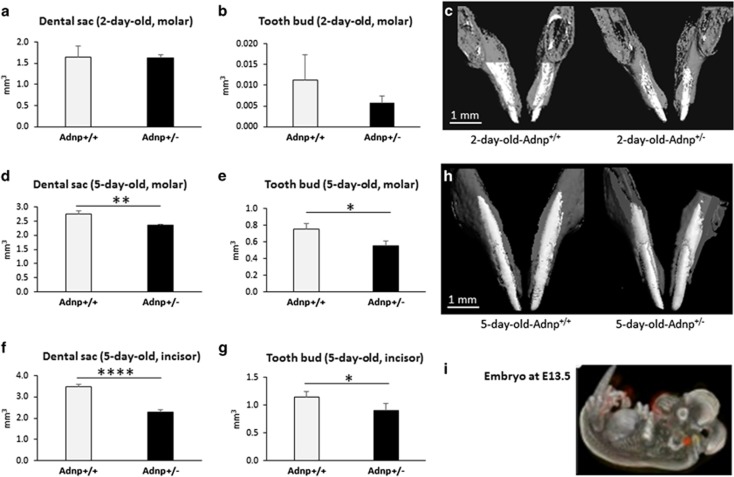
Tooth eruption is delayed in Adnp^+/−^ mice. Two- and five-day-old mouse pup heads were subjected to micro-computed tomography (μCT). (**a–c**) A trend of delayed tooth eruption in 2-day-old Adnp^+/^^−^ pups compared to Adnp^+/+^ pups. (**a**) Dental sac (also defined as the dental follicle contains the developing tooth and its odontogenic organ, *n*=3 mice per group). (**b**) Tooth buds (the tooth bud contains the dental follicle/sac, the enamel organ and the dental papilla, *n*=3 mice per group). (**c**) Picture. (**d–h**) Delayed tooth eruption in 5-day-old Adnp^+/−^ pups as measured both by the size of the dental sac and the tooth buds of molars (**d**, **e**), respectively, *n*=3 mice per group, *P<0.05; **P<0.01, Student's *t*-test) and of incisors (**f**, **g**), respectively, *n*=3 mice per group, *P<0.05; ****P<0.0001, Student's *t*-test). A picture of representative molars at 5 days of age is shown comparing Adnp^+/+^ mice to Adnp^+/−^ mice (**h**). (**i**) Allen Atlas analysis identified ADNP expression in the jaw area. Pictures obtained at embryonic age E13.5 and E15.5 are shown with a clear enrichment of Adnp expression in the jaw area (red, *in situ* hybridizations). ADNP, activity-dependent neuroprotective protein.

To further evaluate the influence of the *Adnp* gene dosage on tooth eruption in mice, we analyzed gene expression in intact Adnp (Adnp^+/+^) and Adnp-haploinsufficient (Adnp^+/−^) mice. Two- and five-day-old mouse pup heads were subjected to μCT (Figure 2). Results suggested normal tooth growth with a trend for a delay in the size of the tooth bud, at 2 days of age, with similar volume of the dental sac (Figures 2a–c). The delay became significant in 5-day-old pups as measured both by the size of the dental sac and the tooth buds of the mandibular molars (Figures 2d and e) and incisors (Figures 2f and g). A picture of representative molars at 5 days of age is shown comparing Adnp^+/+^ mice (Figure 2h) to Adnp^+/−^ mice (Figure 2i). These results further associate Adnp to the regulation of tooth development. It is important to add here that in contrast to humans, who are diphyodonts, mice are monophyodonts.

Our original studies identified ubiquitous expression of ADNP RNA in mice^3^ and human^2^ with substantial expression during embryonic development.^7^ Further investigation using Allen Developing Mouse Brain Atlas (http://developingmouse.brain-map.org) identified specific Adnp mRNA expression suggestive of Adnp innervation of the jaw to regulate tooth eruption (Figure 2j and Supplementary Figure S1).

### ADNP deficiency or mutations regulate bone/tooth morphogenesis genes

Our original studies in mice (Gozes laboratory)^10^ revealed *Bmp1* as one of the major downregulated genes as a consequence of *Adnp* knockout. Bmp1 is required for dentin formation^32^ and represents the first identifiable feature in the crown stage of tooth development. Given ADNP's regulation of Bmp1, we have chosen to investigate (1) does ADNP regulate additional genes associated with bone morphogenesis and (2) how do ASD-related ADNP mutations in children affect gene expression linked with tooth development.

**Figure 3:**
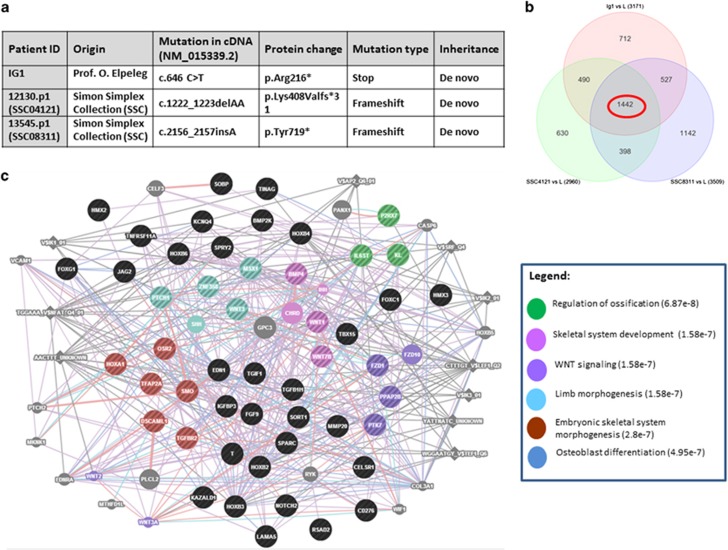
RNAseq comparisons of ADNP-mutated human LCLs' differential expression of gene associated with tooth formation. (**a**) Table of mutated LCLs used for RNAseq analysis. (**b**) Venn diagram of differentially expressed genes (L=control, non-mutated LCL; fold-change difference=2). (**c**) Fifty bone-associated gene networks (genes in black or colored according to pathway legend, Stripped circles represent multiple functions) as presented by the GeneMania software (see Materials and methods) as a consequence of ADNP mutation in LCLs (*P*-value of pathway enrichment is presented in parenthesis). ADNP, activity-dependent neuroprotective protein; LCL, lymphoblastoid cell line; RNAseq, RNA sequencing.

To answer these questions, we have performed complete RNAseq analysis on four representative LCLs including three mutated cell lines (Figure 3a) and one control line. Differentially expressed genes were obtained with fold-change difference >2 or<−2. Our results identified 1442 common genes that were differentially expressed in all the three different LCL-mutated lines compared to the normal LCL line (Figure 3b), including 50 genes functionally associated with bone formation (Supplementary Figure S2). Further network evaluations identified functional and protein–protein interactions between these genes (Figure 3c).

As indicated in our introduction, matrix metalloproteases have an important role in canal eruption, for example, MMP20 has been associated with proper enamel maturation.^33^ Data mining of our RNAseq results showed 2.3–4.9 increase in MMP20 expression in the three ADNP-mutated cell lines tested versus the control line. Interleukin 11 (ref. 19) expression increased by approximately two fold in the p.Arg216* and the p.Tyr719*-ADNP-mutated LCLs, but not in the p.Lys408Valfs*31 LCLs compared to intact ADNP LCLs. RUNX2 decreased in the p.Arg216* LCL (−2.01-fold) and increased in the p.Lys408Valfs*31 LCL (3.29-fold). BMP2 did not show a change in these samples.

**Figure 4:**
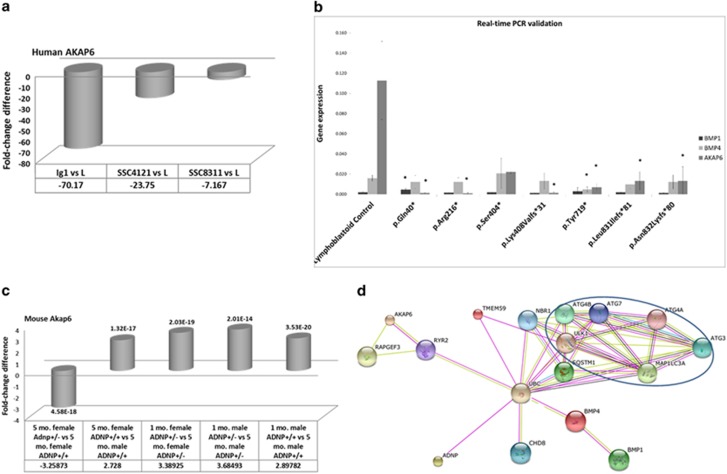
ADNP mutations or knockdown effects on *AKAP6, BMP1* and *BMP4* expression. (**a**) Mutated human *AKAP6* expression compared to control LCLs. (Fold-change difference is shown below the graph. The data represent RNAseq results.). (**b**) The mean and s.d. were calculated from three independent experiments of quantitative RT-PCR. Each mutation bar represents one cell line. In the case of p.Leu831Ilefs*81 and p.Asn832Lysfs*81, two cell lines were available for each of these mutations and gave similar results; therefore, gene expression was calculated as an average for each mutation. Significance was calculated for each gene compared to its expression in the control ADNP-intact line. Student's *t*-test *P<0.05. (**c**) Mutated mouse hippocampal Akap6 expression compared to control LCLs (fold-change difference is shown below figure, RNAseq results from GSE72664 (ref. 4). Numbers above or below boxes represent p(FDR) values (of three replicates). (**d**) STRING figure of ADNP–Autophagy–BMP–CHD8–AKAP6 interactions: discovery of a potential central function relation to UBC. Blue circle indicates genes associated with autophagy. Connections are labeled as indicated in http://string-db.org/. Importantly, pink connections represent experimental evidence, while green lines represent text mining. For more details, see Materials and methods. ADNP, activity-dependent neuroprotective protein; AKAP6, A Kinase Anchor Protein 6; BMP, bone morphogenetic protein; LCL, lymphoblastoid cell line; RNAseq, RNA sequencing; RT-PCR, reverse transcriptase polymerase chain reaction; UBC, ubiquitin C.

Interestingly, when comparing the most differentially regulated genes in the LCLs in the human RNAseq data, A Kinase Anchor Protein 6 (AKAP6), directly associated with bone strength^34^ and cognition,^35^ was found to be markedly downregulated in the three ADNP-mutated LCLs compared to the control line (Figure 4a). These results were verified by quantitative reverse transcriptase PCR (RT-PCR), extending the finding to the currently available ADNP mutant LCLs at the Gozes laboratory (Figure 4b). Results showed that in the p.Gln40*, p.Arg216*, p.Tyr719*, p.Leu831Ilefs*81 and p.Asn832Lysfs*81 ADNP-mutated LCLs, *AKAP6* was downregulated; in p.Ser404*-ADNP-mutated LCL only an apparent downregulation was evident. Further PCR analysis was carried out for *BMP1* and *BMP4*, showing an increase in *BMP1* expression in the p.Gln40*-ADNP-mutated LCL, which we have previously shown to be downregulated in Adnp knockout embryos.^10^ Furthermore, *BMP4* was significantly decreased in the p.Tyr719*-ADNP-mutated LCL. The significance of the results is strengthened by genome-wide transcriptomic studies of human LCLs (12 independent lines) showing limited variations in *AKAP6* (measurements of 2.87+0.543, arbitrary numbers) and *BMP4* (3.67+0.307).^36^ Further verification used another independent control LCL (Supplementary Figure S3).

A comparison to our previous RNAseq data in mice (hippocampal samples),^4^ which was performed concomitantly with the human study, indicated that among the most differentially expressed genes in the LCLs as a consequence of the human mutation, *Akap6* showed a relatively high number of total reads (101 126 reads) in the mouse. Further analysis in the Adnp^+/−^ mice (Figure 4c) revealed differential expression in 5-month-old female mice with a reduction in the Adnp^+/−^ mice compared to Adnp^+/+^ mice (fold-change=−3.2; p(FDR)=4.58E^−^^18^), a genotype-related regulation, similar to the reduction observed as a consequence of the different mutations in human LCLs. These results suggest that the mutations in ADNP, at least in this case, are associated with a loss of function. Furthermore, a difference between 5-month-old female Adnp^+/+^ and 5-month-old male Adnp^+/+^ mice in the expression of *Akap6* was observed, with higher expression in the Adnp-intact females compared to male mice (fold-change=2.7; p(FDR)=1.32E^−^^17^). A consistent age-related increase (fold-change>3; p(FDR)<E^−14^) in *Akap6* expression was observed in 1-month versus 5-month-old mice (except in female Adnp^+/+^, Figure 4c).

### The ubiquitin C connection and other potential contributing factors

To further analyze ADNP connection to AKAP6, we have created the STRING network (Figure 4d) linking autophagy-associated proteins to ADNP through ubiquitin C (UBC),^37^ which, in turn, connects to AKAP6 (ref. 38) through the ryanodine receptor (RyR)/calcium release channel (RYR2),^39^ with calcium channels being directly related to ADNP function.^4^ In addition, UBC is directly connected to BMP4 (ref. 40) and to CHD8,^37^ with the latter being a key gene mutated in autism.^41, 42^ We focused on autophagy, as we have previously shown a direct binding of ADNP to the microtubule-associated protein 1 light chain 3 (LC3B)^6^ as well as changes in Becl1 expression resulting from Adnp deficiency.^4^ Interestingly, RYR2 is required for cardiac muscle excitation–contraction coupling^38^ and ADNP has been shown to be required for heart development in mouse^10^ and men.^12^ UBC was shown here to be a central point for protein–protein interaction (Please see Supplementary Information for additional supporting data mining).

Furthermore, it is possible that low muscle tone is also a contributing factor as our original findings identified Myosin light chain 2 (*yl2*) as a major gene directly regulated by Adnp in mice, in a sex- and age-dependent manner.^4, 10^ Our current RNAseq data identified Myosin, Light Chain 9, Regulatory (*MYL9*) as a major downregulated gene (by 40- to 60-fold) in LCLs from ADNP-mutated p.Lys408Valfs*31 and ADNP-mutated p.Arg216*, respectively.

## Discussion

Here we implicated early deciduous teething as a strong early diagnostic marker for potential ADNP mutation, and showed that ADNP dysregulation affects tooth eruption in mice (monophyodonts) and men (diphyodont). According to the American Dental Association,^31^ at 12 months, children rarely display more than the incisor teeth. Surprisingly, unlike a multiplicity of syndromes showing delayed or failure of dentition,^15^ the ADNP-mutated children show early deciduous dentition, which agrees in part with the intricate regulation of gene expression exerted by ADNP. As the mutated-ADNP phenotype is close in features to ASD,^41, 42^ early or altered deciduous tooth eruption timing is of interest to a range of undiagnosed children within the autism/mentally challenged spectrum. These findings may help identify children at risk at 1 year of age, a time that is extremely early in autism diagnosis.^43^ Interestingly, the eruption timing for the first deciduous tooth correlates with the first permanent tooth eruption,^44^ and we show premature deciduous tooth-shedding and early permanent tooth eruption in the ADNP-mutated children.

Looking at the chronology/sequence of eruption of deciduous teeth in Spanish children, the first tooth to erupt was the lower right central incisor at 10.96±1.88 months, and the last was the upper left second molar, at 33.24±4.35 months; symmetry was found in the eruption of the deciduous teeth. Thus, in an unbiased population, only the mandibular central incisors could be found at 1 year of age.^30^ In this study, statistically significant gender differences were found, and while those were considered clinically irrelevant, sex differences were observed before for ADNP expression.^4, 25, 45^ On the basis of twin and family studies, the timing of deciduous tooth eruption is highly heritable, with estimates typically exceeding 80%. Genome-wide association study of primary dentition identified pleiotropic loci associated with height and craniofacial distances, including BMP4.^14^ Our findings of early deciduous teething as well as the previously observed recurrent facial dysmorphic features in the ADNP-mutated children^11^ and our (Gozes) previous discovery of generally delayed development in Adnp^+/−^ mice^24^ tie ADNP to BMP regulation. Furthermore, among the genes found in the functional analysis (Supplementary Figure S2 and Figure 4d), signaling of Bmp4 that may be associated with the ADNP mutations suppresses tooth developmental inhibitors, and changes in its expression may change teething time.^46^ Together, with our previous discovery of ADNP-regulating BMP1,^10^ the current findings indicate that ADNP is regulating the *BMP* gene family associated with tooth formation.

Our studies showed altered odontogenesis in both ADNP-mutated children (diphyodonts) and Adnp-haploinsufficient mice (monophyodonts). However, the result was opposite with ADNP mutation in children showing early deciduous dentition in contrast to an apparent delayed eruption of the permanent teeth in Adnp^+/−^ mice. This may be construed as a study limitation; however, it should be noted that the most common model for the study of odontogenesis, the mouse, possesses only one generation (monophyodont) and two classes of teeth.^47^ It should also be noted that odontogenesis disturbance in humans may result in opposite eruption timing (delayed/early) in the permanent versus deciduous dentitions as reported in patients with orofacial clefts.^48^ Thus, despite the differences in manner and timing, our results suggest that ADNP regulates teething in both mice and men.

Interestingly, our original discovery of ADNP was based on a search for neuroprotective proteins regulated by the major regulatory vasoactive intestinal peptide.^3, 49, 50^ Considering the high homology shared by vasoactive intestinal peptide and pituitary adenylate cyclase-activating polypeptide (PACAP), we have tested and shown PACAP regulation of ADNP expression,^51^ and these findings were extended to show co-localization of the PACAP) type 1 receptor (PAC1) with Adnp in the mouse brain.^52, 53^ Indeed, immunoreactive PACAP nerve fibers were identified in rat and human tooth pulp.^54^ Further studies on PACAP-knockout mice revealed that the dentin was significantly thinner in the molars of PACAP-deficient mice compared to wild-type animals.^55, 56^ Our current studies have not revealed mineral density changes in the Adnp-deficient pups, suggesting converging and disparate pathways for ADNP and PACAP.

Regarding ADNP gene regulation, a study limitation in our RNAseq analysis was the paucity of samples (only three mutated LCLs and one intact LCL, Figure 3). To overcome this limitation, we have also verified some of the data in additional mutated LCLs (Figure 4) and compared our human data with mouse data (Figure 4).

Our String analysis (Figure 4d) puts the ubiquitin system (UBC) in a central point for ADNP regulatory pathways, and in that respect, Angelman syndrome is caused by ubiquitin protein ligase E3A mutations. Angelman syndrome (affecting ~1:15 000 individuals) is characterized by motor dysfunction, severe mental retardation, speech impairment, frequent seizures, hyperactivity and a high prevalence of autism.^57^ Angelman syndrome shares similarities to the ADNP syndrome, and is part of the differential diagnosis of the syndrome.^12^ Importantly, ubiquitination is not only linked to Angelman syndrome, but also among others, to Down syndrome, including five genes involved in the ubiquitination pathways on chromosome 21.^58^ Of note, Down syndrome was also associated with delayed teething in a sex-dependent manner.^59^

Our findings identifying early deciduous teething, as a noninvasive simple diagnostic measure of ADNP mutation, will support targeted sequencing to pinpoint the mutation at an early phase of life. With the recent finding of ADNP as being one of a small group of genes, including CHD8 and SHANK3 that appear to lead to autism in a substantial proportion of cases,^60^ the significance of our current results is enhanced. Furthermore, early diagnosis should help navigate to potential future personalized intervention.

### Data and materials availability

RNAseq results have been uploaded on GEO (GSE81268). Adnp-haploinsufficient mice must be obtained through material transfer agreements (MTAs).

## Figures and Tables

**Table 1:**
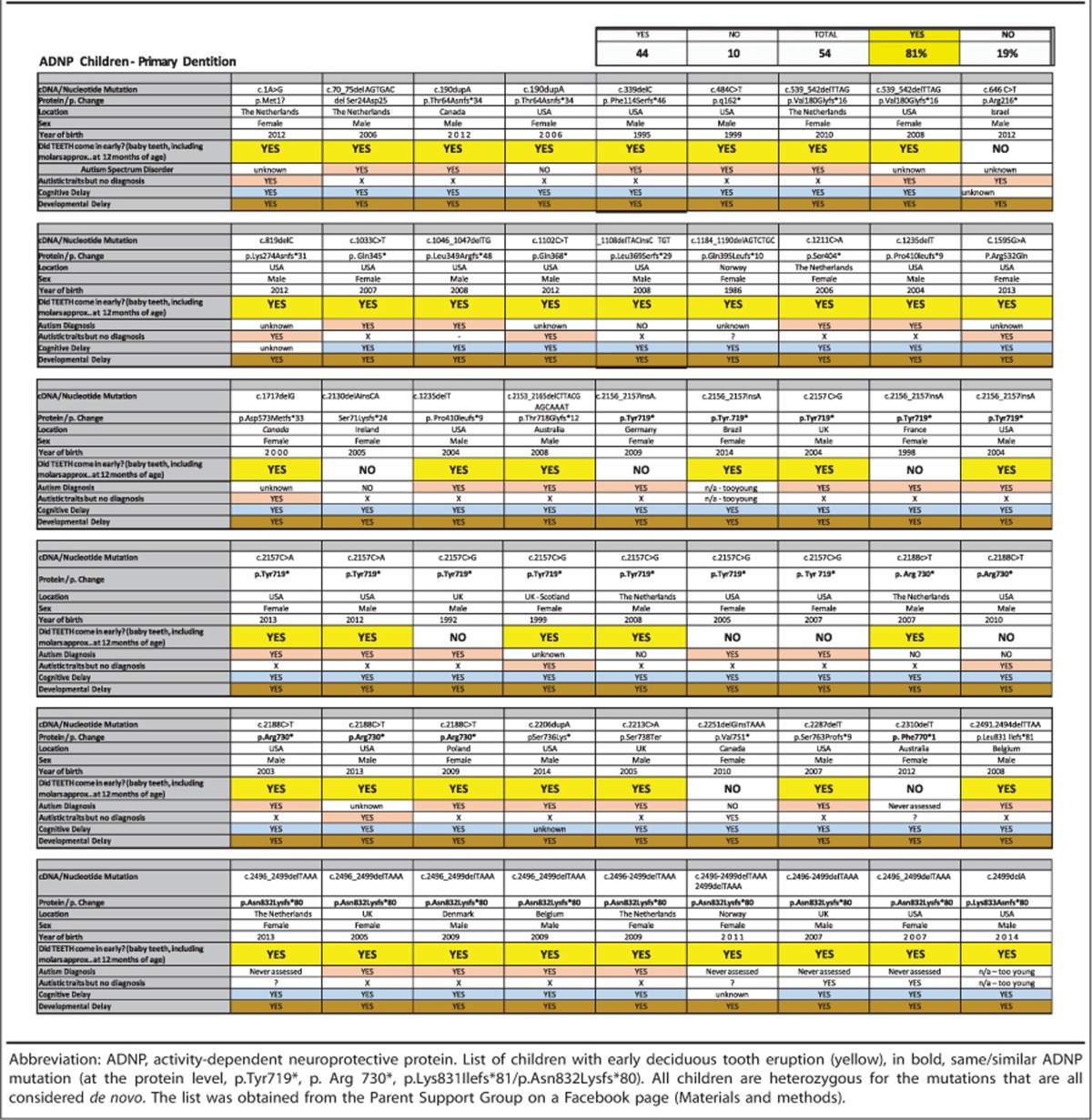
Deciduous tooth eruption is early in ADNP-mutated children
