# Premature primary tooth eruption in cognitive/motor-delayed ADNP-mutated children

**DOI:** 10.1038/tp.2017.128

**Published:** 2017-07-04

**Authors:** I Gozes, A Van Dijck, G Hacohen-Kleiman, I Grigg, G Karmon, E Giladi, M Eger, Y Gabet, M Pasmanik-Chor, E Cappuyns, O Elpeleg, R F Kooy, S Bedrosian-Sermone

**Correction to**: *Translational Psychiatry* (2017) **7**, e1043; doi:10.1038/tp.2017.27; published online 21 February 2017

Several entries in Table 1 use protein change annotations that do not comply with Human Genome Variation Society nomenclature. The nomenclature corrections (obtained from https://mutalyzer.nl/) are listed below. Most of the changes are a result of one amino acid to the truncated protein complying with the consensus nomenclature. The revised version of the table provided here reflects the corrections.

Panel 1

c.70_75del AGTGAC was changed to p.Ser24_Asp25del instead of del Ser24Asp25.
c.190dupA was changed to p.Thr64Asnfs*35 instead of p.Thr64Asnfs*34.
c.339delC was changed to p.Phe114Serfs*47 instead of p. Phe114Serfs*46.
c.484C>T was changed to p.Gln162* instead of p.q162*.
c.539_542delTTAG was changed to p.Val180Glyfs*17 instead of p.Val180Glyfs*16.

Panel 2

c.1046_1047delTG was changed to p.Leu349Argfs*49 instead of p.Leu349Argfs*48.
c.1106_1108delTACinsCTGT was changed to p.Leu369Serfs*30 instead of p.Leu369Serfs*29.
c.1184_1190delAGTCTGC was changed to p.Gln395Leufs*11 instead of p.Gln395Leufs*10.
c.1235delT was changed to p.Leu412Profs*10 instead of p. Pro410leufs*9.

Panel 3

c.2130delAinsCA was changed to c.2129dupC. Also, a typographical error in protein annotation was corrected: Ser71Lysfs*24 should have been p.Ser711Lysfs*24.
Column 3 (c.1235delT) was deleted entirely. It was a duplicate entry instead of the information for a child who was inadvertently omitted. In the revised table, the entry for the omitted child appears in the last column (see correction 19). The order of the children has been preserved, and the error did not alter the reported number of children who exhibited early tooth eruption and hence did not affect the statistical analysis.
c.2153_2165delCTTACGAGCAAAT was changed to p.Thr718Argfs*6 instead of p.Thr718Glyfs*12.

Panel 5

c.2206dupA was changed to p.Ser736Lysfs*2 instead of pSer736Lys*.
c.2213C>A was changed to p.Ser738* instead of p.Ser738Ter.
c.2310delT was changed to p.Leu771* instead of p. Phe770*1.
c.2491_2494delTTAA was changed to p.Leu831Ilefs*82 instead of p.Leu831 llefs*81.

Panel 6

c.2496_2499delTAAA was changed to p.Asn832Lysfs*81 instead of p.Asn832Lysfs*80.
c.2499delA was changed to p.Val834Serfs*80 instead of p.Lys833Asnfs*80.
The information for the omitted child noted in correction 11 has been added to the last column (c.2865_2868delTGAG).
In column 6, the duplicate nucleotide sequence has been deleted.

## Figures and Tables

**Table 1:**
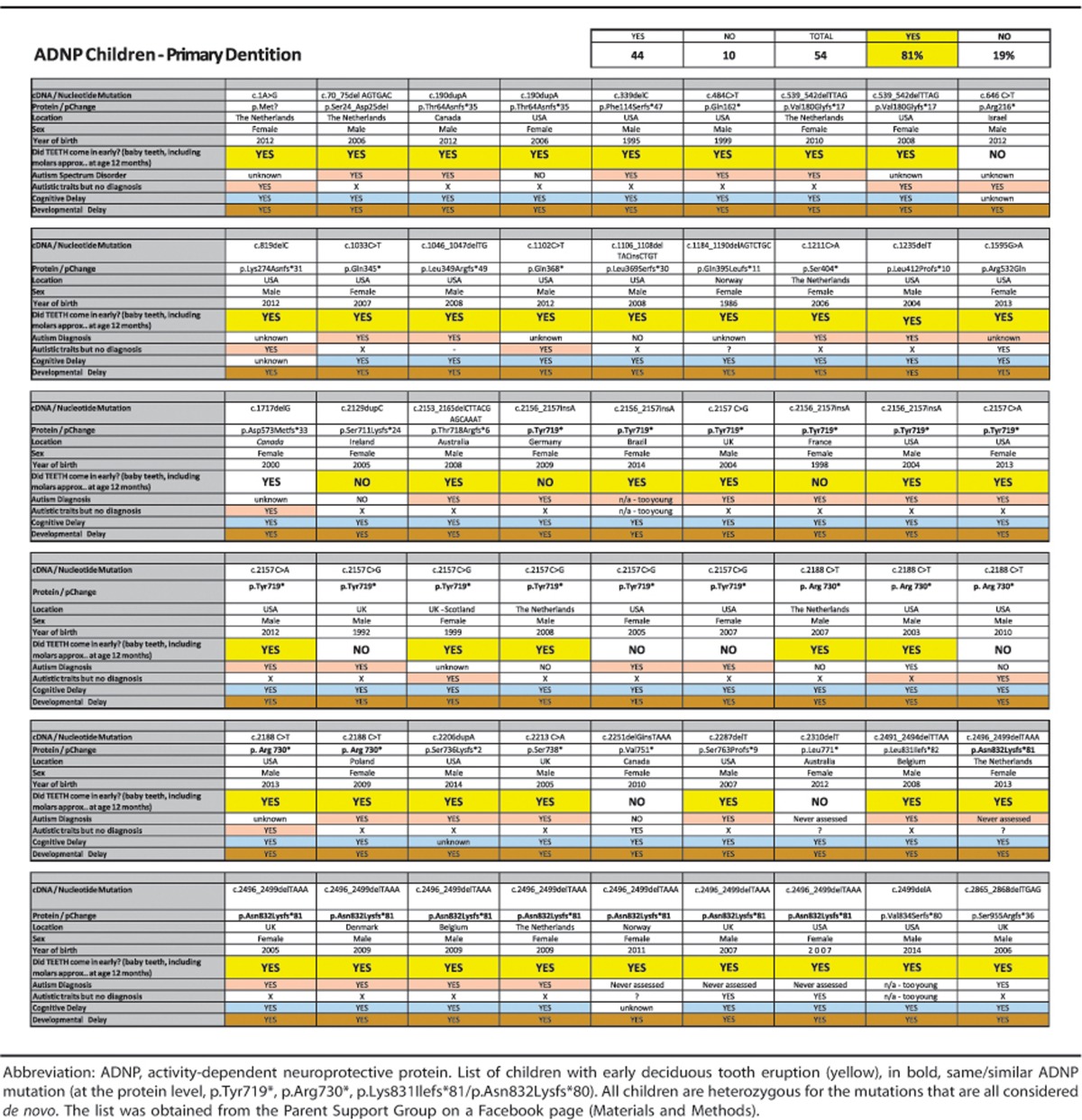
Deciduous tooth eruption is early in ADNP-mutated children

