# Supplementary material for: Premature primary tooth eruption in cognitive/motor-delayed ADNP-mutated children
Source: Transl Psychiatry. 2017 Feb 21;7(2):e1043–. doi: 10.1038/tp.2017.27 (PMC5438031; doi:10.1038/tp.2017.27)
Supplement: Supplementary Information [file tp201727x1.pdf]

Supplemental

Premature Primary Tooth Eruption in Cognitive/Motor Delayed ADNP-Mutated Children

I. Gozes[1,2]*, A. Van Dijck[3], G. Hacohen Kleiman[1,2], I. Grigg[1,2], G. Karmon[1,2], E. Giladi[1,2], M. Eger[2,4], Y. Gabet[2,4], M. Pasmanik-Chor[2,5], E. Cappuyns[3], O. Elpeleg[6], R.F. Kooy[3], S. Bedrosian Sermone[7]

[1]The Lily and Avraham Gildor Chair for the Investigation of Growth Factors; The Elton Laboratory for Neuroendocrinology; Department of Human Molecular Genetics and Biochemistry, Sackler Faculty of Medicine, Sagol School of Neuroscience and Adams Super Center for Brain Studies.

[2]Tel Aviv University, Tel Aviv 69978, Israel.

[3]Department of Medical Genetics, University and University Hospital of Antwerp, Antwerp, Belgium.

[4]Department of Anatomy and Anthropology, Sackler Faculty of Medicine, Sagol School of Neuroscience.

[5]The Bioinformatics Unit, George S. Wise Faculty of Life Sciences.

[6]Monique and Jacques Roboh Department of Genetic, Hadassah Hebrew University Medical Center, Jerusalem, Israel.

[7]ADNP kids Research Foundation, Brush Prairie, WA, USA.

*To whom correspondence should be addressed: igozes@post.tau.ac.il

Supplemental methods:

RNA seq

In short, RNA was extracted using TRI reagent (T9242, Sigma-Aldrich, MO, USA), followed by mRNA library construction (Customary Illumina TruSeq™ RNA Sample Prep Kit v2 protocol (catalog No. RS-122-2001). RNA libraries were pooled and sequenced on two 50 bp single-read HiSeq 2500 lane with V4 reagents at the Technion's Genomic center, Israel (http://www.illumina.com/systems/hiseq_2500_1500/kits.html). Mutated samples were compared to normal LCLs (FastQ files) using Partek® Flow (version 4.0; Copyright ©; 2015 Partek Inc., St. Louis, MO, USA; http://www.partek.com/) pipeline. Bases with quality score less than 20 were omitted, followed by adapter trimming. Alignment was performed using Bowtie2 and quantified to GRCh38 genome using Partek expectation maximization (EM) algorithm, followed by. Gene Specific Analysis (GSA) of reads per kilobase of exon per million fragments mapped (RPKMs). Differentially expressed genes were selected using fold-change cutoff = 2.

Supplemental Results and Figures:

Allen Developing Mouse Brain Atlas [Internet]. Available from: http://developingmouse.brain-map.org identified specific Adnp mRNA expression suggestive of Adnp innervation of the jaw (Supplemental Fig. S1).

Supplemental Fig. 1. Allen Atlas analysis identified ADNP expression in the jaw areas.

Pictures obtained by different imaging technologies at representing embryonic ages E13.5 and E15.5 are shown with a clear enrichment of Adnp expression in the jaw areas (red, in situ hybridizations).

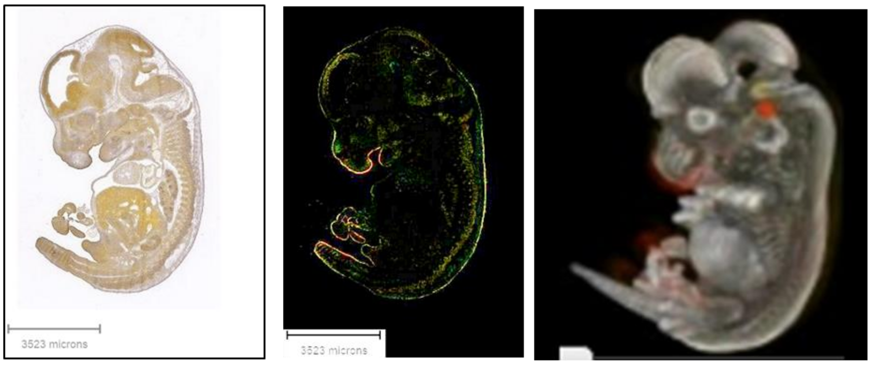

Age E13.5

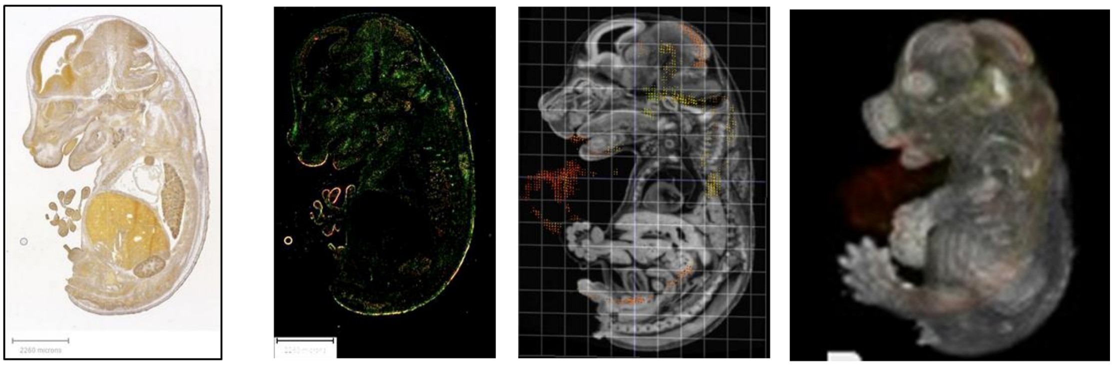

Age E15.5

Our results further identified 1442 common genes that were differentially expressed in all the three different LCL mutated lines tested compared to the normal LCL line (Fig. 3B, main text). Of these a total of 50 genes were functionally associated with bone formation (Supplemental Fig. S2), although not all were enriched with significant p[FDR] values).

Supplemental Fig. 2. Function enrichment of bone\tooth related functions (DAVID function enrichment tool, see Materials and Methods) of 1442 common

differentially expressed human genes. 50 genes were associated with functions related to bone \tooth development and regulation.

Function enrichment (from DAVID) of 1442 common differentially expressed (DE) human genes:

Gene ontology	p-value	genes in function
regulation of bio-mineral formation	0.06	BMP4, P2RX7, MMP20, KL, BMP2K, CD276
positive regulation of bone mineralization	0.12	BMP4, P2RX7, KL, CD276, BMP2K
bone development	0.47	BMP4, HOXB4, SMO, TNFRSF11A, FGF9, KAZALD1, SORT1, FOXC1, SPARC, IGFBP3
regulation of ossification	0.16	BMP4, P2RX7, WNT7B, KL, IL6ST, BMP2K, CD276, FZD1, RSAD2
embryonic morphogenesis	0.003	HMX2, FGF9, EDN1, PTK7, JAG2, DSCAML1, SOBP, T, HOXA1, WNT1, SPRY2, KCNQ4, OSR2, WNT3, PPAP2B, BMP4, TBX15, TGFBR2, CELSR1, ZNF358, TINAG, HOXB3, NOTCH2, HOXB4, HOXB2, MSX1, LAMA5, HOXB6, FOXG1, TGIF1, TFAP2A, FOXC1, PTCH1, TGFB1I1, HMX3
embryonic skeletal system morphogenesis	0.04	HOXB3, HOXB4, HOXB2, OSR2, TBX15, HOXB6, TGFBR2, DSCAML1, TFAP2A

Supplemental Fig. 3. ADNP mutations on AKAP6 and BMP1 expression (an additional control LCL compared to two mutated LCLS

Mean and standard deviation were calculated from three independent experiments of quantitative RT-PCR. Each mutation bar represents one cell-line. Significance was calculated for each gene compared to its expression in the control ADNP-intact line. Student t-test *p<0.05.

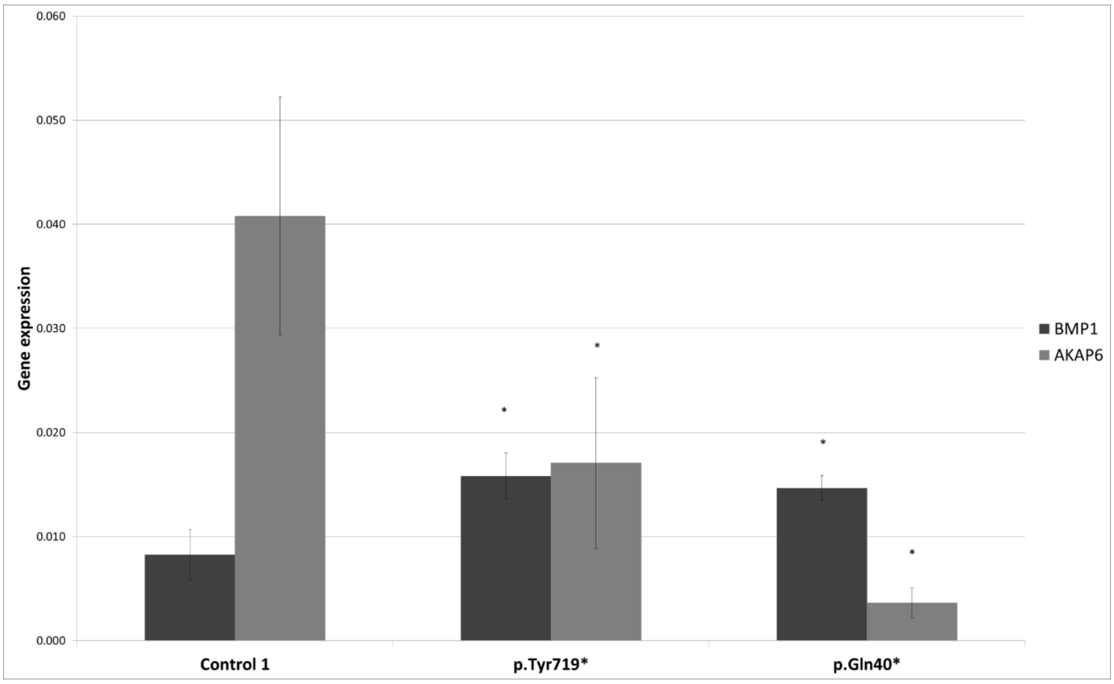

The ubiquitin C (UBC) connection: additional data mining

Looking further at transcripts that showed highly significant differences in LCLs from mutated ADNP children and compared to normal individuals identified the Ubiquitin Specific Peptidase 9, Y-Linked (USP9Y) as the most significantly upregulated transcript in the ADNP mutated p.Arg216* LCL (~1500-fold increase). A similar finding was also observed in the p.Tyr719* mutated LCL (~3520-fold increase). Notably, the control LCL and the two mutated LCLs are males; the third mutated line tested is female, not showing an impact on the Y-linked transcript. These findings also place the ubiquitin system at a central point of ADNP mutation deregulation, probably associated with early dentition .

In an independent study (Helsmoortel, PhD thesis, submitted) five/six patients and eight control LCLs were analyzed on HumanHT-12 v4 ExpressionBeadChip (Illumina) and the top differentially expressed genes included UCHL1, encoding the ubiquitin carboxyl-terminal hydrolase isoenzyme L1, again, linking ADNP and ADNP mutations to the ubiquitin system.
